# Direct Production of a Hyperpolarized Metabolite on a Microfluidic Chip

**DOI:** 10.1021/acs.analchem.1c05030

**Published:** 2022-02-11

**Authors:** Sylwia J. Barker, Laurynas Dagys, William Hale, Barbara Ripka, James Eills, Manvendra Sharma, Malcolm H. Levitt, Marcel Utz

**Affiliations:** † School of Chemistry, University of Southampton, Southampton SO17 1BJ, United Kingdom; ‡ Institute for Physics, Johannes Gutenberg University, D-55090 Mainz, Germany; ¶ Department of Chemistry, University of Florida, Gainesville 32611, United States; § GSI Helmholtzzentrum für Schwerionenforschung GmbH, Helmholtz-Institut Mainz, 55128 Mainz, Germany

## Abstract

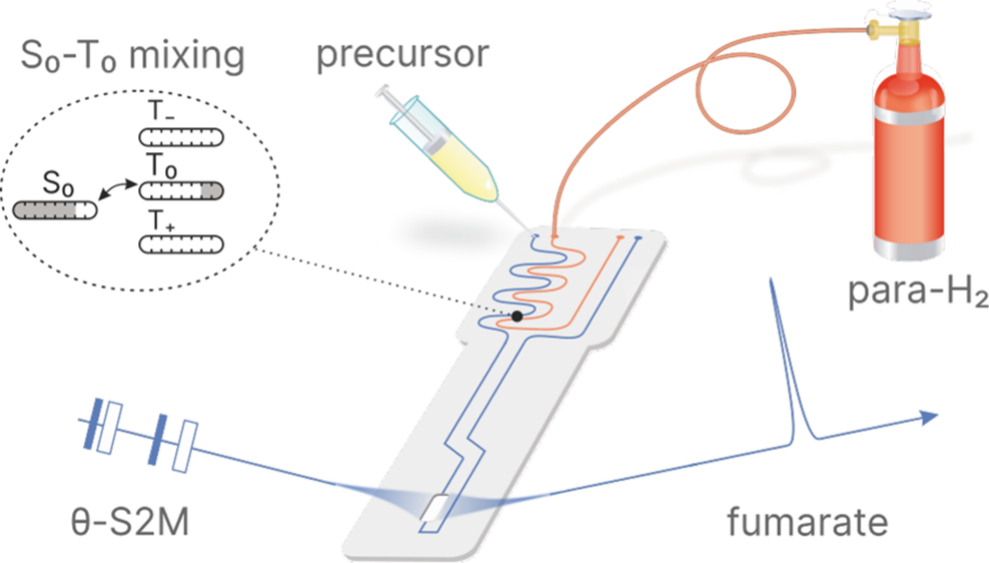

Microfluidic systems hold great potential for the study of live microscopic cultures of cells, tissue samples, and small organisms. Integration of hyperpolarization would enable quantitative studies of metabolism in such volume limited systems by high-resolution NMR spectroscopy. We demonstrate, for the first time, the integrated generation and detection of a hyperpolarized metabolite on a microfluidic chip. The metabolite [1-^13^C]fumarate is produced in a nuclear hyperpolarized form by (i) introducing para-enriched hydrogen into the solution by diffusion through a polymer membrane, (ii) reaction with a substrate in the presence of a ruthenium-based catalyst, and (iii) conversion of the singlet-polarized reaction product into a magnetized form by the application of a radiofrequency pulse sequence, all on the same microfluidic chip. The microfluidic device delivers a continuous flow of hyperpolarized material at the 2.5 μL/min scale, with a polarization level of 4%. We demonstrate two methods for mitigating singlet–triplet mixing effects which otherwise reduce the achieved polarization level.

## Introduction

Nuclear magnetic resonance (NMR) is a versatile spectroscopic technique, well-suited for noninvasively probing complex chemical systems and their dynamic behavior. The sensitivity of NMR is limited by the polarization of nuclear spins, which is small in thermal equilibrium even at the largest available magnetic fields. Hyperpolarization methods such as parahydrogen-induced polarization (PHIP)^1−5^ can produce much larger spin alignments in special cases, offering several orders of magnitude enhancements in sensitivity. This is particularly attractive in the context of microfluidic lab-on-a-chip (LoC) devices, where sample volumes are typically of the order of nL to μL.^6^ Such LoC are versatile platforms on which chemical and biological systems can be studied under precisely controlled and reproducible conditions. LoC systems are commonly used as scaffolds for cell^7−11^ and organ^12−15^ cultures, providing valuable models for supporting the development of diagnostics,^16,17^ therapies^12^ and drug safety testing^18,19^ but also for chemical reaction monitoring.^20^ While state-of-the-art micro-NMR probes can provide ^1^H NMR detection sensitivities of around  for microliter-scale samples in a 14 T magnet at thermal equilibrium,^21^ this can be improved into the range of  by PHIP.^22^ Like other hyperpolarization methods, PHIP requires specific chemical processes and spin manipulations to produce hyperpolarized species. LoC devices can be used to implement some or all of these processes, thus offering the possibility to integrate production and application of hyperpolarized species in a single, compact platform.

PHIP is conventionally implemented by bubbling hydrogen gas enriched in the para spin isomer through a solution containing a suitable substrate and a catalyst, either directly at high magnetic field (PASADENA experiments)^23^ or outside of the magnet at low (μT) fields, followed by an adiabatic increase of the magnetic field (ALTADENA experiments).^24^ Such experiments are effective but quite difficult to repeat accurately. This complicates systematic studies of the interplay between reaction kinetics and nuclear spin relaxation processes. As we have recently shown, microfluidic implementation of PHIP at high field allows delivery of the hydrogen gas by diffusion through a membrane, such that no bubbling is required.^22^ Experiments can therefore be carried out under continuous flow, with a stable stationary level of hyperpolarization established in the chip. This can be exploited for hyperpolarized multidimensional NMR experiments, which require superposition of many transients that must maintain a high level of consistency.

In the following, we use the same approach to probe the formation of hyperpolarized [1-^13^C]fumarate from [1-^13^C]disodium acetylenedicarboxylate in an aqueous solution. To our knowledge, this is the first demonstration of PHIP-hyperpolarized metabolite production in a microfluidic device. Hyperpolarized fumarate is widely used as a contrast agent for in vivo detection of necrosis.^25−35^ While the current implementation is not yet ready for use with biological systems due to the presence of the catalyst and other residues, the stability of the microfluidic implementation allows systematic studies of complex kinetic effects.

In this work, we generate and observe solutions of [1-^13^C]fumarate formed via trans-hydrogenative PHIP in a microfluidic chip under continuous-flow conditions, performing the chemical reaction in one part of the chip and NMR detection in another. The operation of this device has been discussed in detail elsewhere.^22,35^ Briefly, all of our experiments are performed inside of a high field NMR spectrometer where the reaction solution containing the precursor and the catalyst is delivered to the chip via a syringe pump. Parahydrogen is delivered through a separate channel and diffuses through the PDMS membrane to dissolve into the precursor solution; hence, the hydrogenation reaction takes place in the chip.

Microfluidic technology provides a convenient platform for studying hyperpolarized NMR experiments for the following reasons:^6^

1. The results are more reproducible since hydrogen is brought into solution via diffusion through a membrane, which is less erratic than bubbling or shaking.^22,36−38^
2. The reaction kinetics and relaxation properties do not vary between or during experiments since a steady-state can be established between the rate of reaction and relaxation, and this can be finely tuned by, e.g., varying the flow rates used.^22,36−38^
3. The low volumes used in microfluidics (in this work a few microliters) makes it more practical to work with expensive or rare samples.
4. Since fresh reaction solution is continuously provided to the detection chamber, the samples do not need to be replaced between experiments.^22,37^
5. Bringing the hyperpolarization step close to the point of detection minimizes the signal losses due to relaxation.

Singlet–triplet mixing has been reported to hinder the achievable polarization of [1-^13^C]fumarate at high field.^33,39,40^ Using our PHIP-on-a-chip system, we quantify how effectively two different RF pulse methods mitigate the problem of ST mixing and support our finding with computational spin dynamics simulations. We present quantitative data on the kinetics and yield of [1-^13^C]fumarate from [1-^13^C]disodium acetylenedicarboxylate in a microfluidic device.

## Background

**Figure 1:**
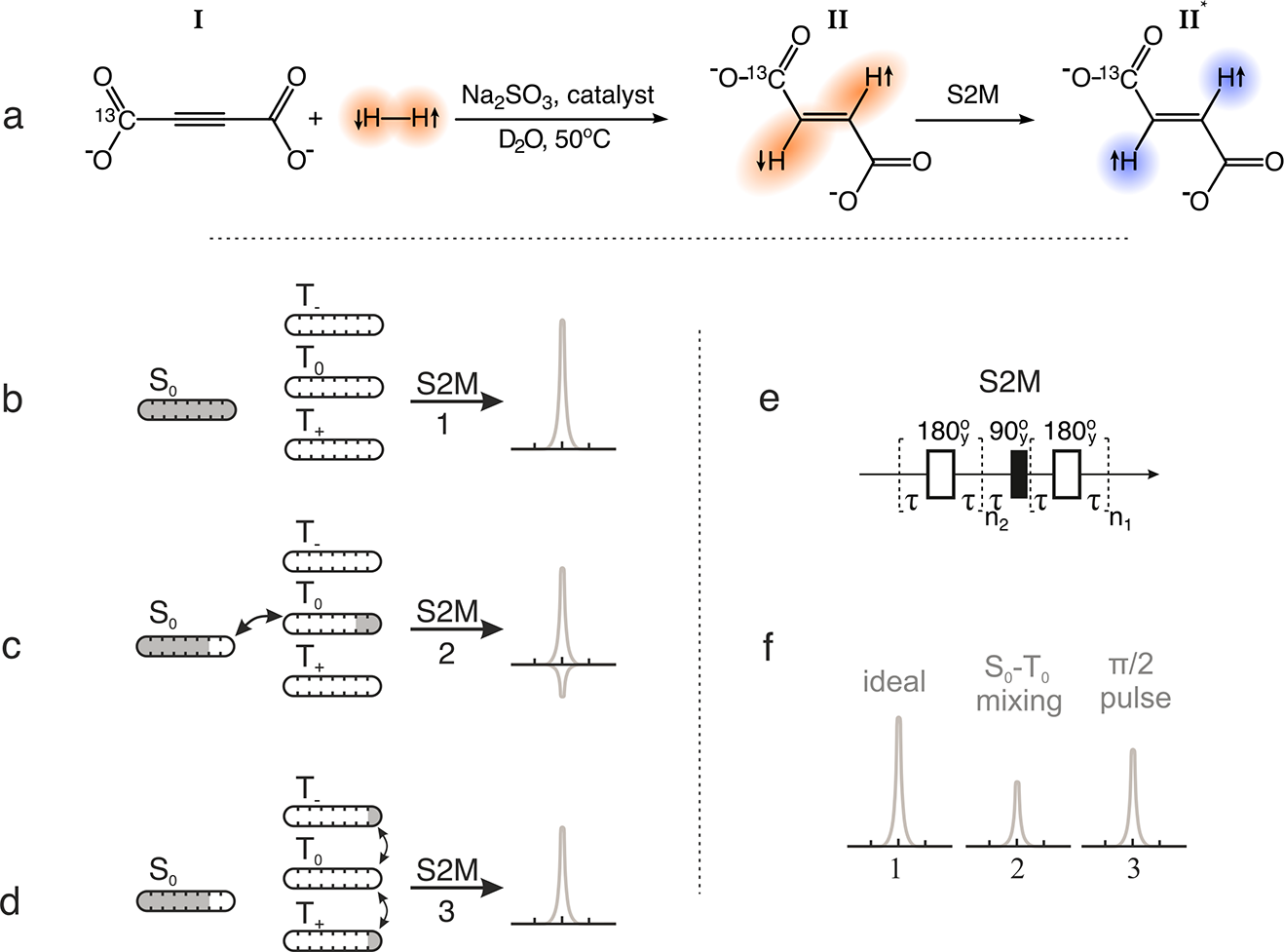
(a) Scheme of the reaction investigated in this work. [1-^13^C]disodium acetylenedicarboxylate labeled as molecule **I** reacts with parahydrogen in the presence of sodium sulfite and the catalyst [RuCp*(CH_3_CN)_3_]PF_6_ in D_2_O at 50 °C. The reaction results in a production of [1-^13^C]disodium fumarate, molecule **II**, with the two protons in a singlet state. Application of the S2M pulse sequence converts the singlet state into a state that is magnetic and hence observable, molecule II*. (b) Illustration of an ideal case where no ST mixing occurs; only the |*S*_0_⟩ state is populated. (c) A case where ST mixing occurs leading to a leak of |*S*_0_⟩ state population to the |*T*_0_⟩ state. (d) A case where the ST mixing is negated by applying a purge pulse prior the S2M, which distributes the population of the |*T*_0_⟩ state to |*T*_+_⟩ and |*T*_–_⟩ states. (e) S2M pulse sequence converts the singlet order into observable hyperpolarized magnetization. The optimal parameters for this molecular system are τ = 15.6 ms, *n*_2_ = 14, *n*_1_ = 7. (f) Predicted signal intensities for three different scenarios.

The reaction shown in Figure 1a produces [1-^13^C]fumarate **II** by hydrogenation of [1-^13^C]disodium acetylenedicarboxylate **I** with parahydrogen in the presence of a ruthenium catalyst. The slight magnetic inequivalence due to the difference in ^1^H–^13^C  *J*-couplings makes it possible to convert the singlet order into observable hyperpolarized magnetization through the use of RF pulse sequences. In this work we use the singlet-to-magnetization (S2M) pulse sequence for this task (see Figure 1e), which is robust against field inhomogeneities in contrast to alternative methods.^41^ This is important because magnetic field inhomogeneities are present in the chip due to the differences in magnetic susceptibility of the chip and the solvent.^42^ Applying this sequence after the chemical reaction with parahydrogen results in high magnetization of the two protons giving rise to a hyperpolarized substance II*.

The polarization that is generated on the target molecules can be attenuated by singlet–triplet (ST) mixing (sometimes called ST leakage) .^43^ The hydrogen molecules can form intermediate hydride species with the catalyst metal center, where the two hydrogen atoms take up inequivalent positions, such that they experience a chemical shift difference at high field. If the lifetime of this intermediate complex is long enough, there can be a significant leakage from the H_2_ proton singlet state (|*S*_0_⟩) to the central triplet state (|*T*_0_⟩), which generally reduces the resulting PHIP signals.^43−45^ The S2M sequence converts both the |*S*_0_⟩ and the |*T*_0_⟩ states to magnetization but with opposite phases. The population of the |*T*_0_⟩ state therefore *reduces* the resulting NMR signal, as illustrated in Figure 1c. This process sometimes gives rise to a partially negative line (PNL) in the ^1^H NMR spectra.^46^ It is also known to occur in non-hydrogenative PHIP experiments and has been noted to give rise to “spontaneous” polarization on the target molecules,^47^ although generally ST mixing is undesirable.

Two methods have been shown to suppress ST mixing: spin locking on the ^1^H hydride resonance during the chemical reaction^39,46,48−56^ and applying a hard π/2 purge pulse to deplete the |*T*_0_⟩ state prior to the polarization transfer step.^46,52,55−59^ These two methods are illustrated in Figure 1d.

As we show in the following, the study of ST mixing is greatly facilitated by microfluidic PHIP, since instabilities associated with bubbling experiments are avoided. Additionally, since hydrogenative PHIP relies on irreversible chemical reactions, the chemical kinetics influence the observed spectra, and the sample under study would need to be replaced upon the reaction reaching completion. This is a particular issue if the samples are scarce or expensive due to isotopic enrichment. Finally, since hyperpolarized nuclei are in a nonequilibrium state, the NMR signals relax on a time scale of seconds to tens of seconds, unique to each molecular species and nuclear spin site, which can convolute the observed results. This is especially problematic if the signals relax quickly compared to the time it takes for a shaken tube to be placed in the NMR magnet or for bubbles to settle in solution.

## Materials and Methods

All experiments were performed in a 11.7 T magnet using a Bruker AVANCE III spectrometer system. The NMR experiments were performed with a custom-built probe delivering ^1^H RF pulses of 125 kHz amplitude.^21^^1^H spectra were collected with a 16 ppm spectral width and 8 k point density.

Para-enriched hydrogen gas (gas purity 99.995%) was continuously produced by a Bruker parahydrogen generator BPHG90, with a specified parahydrogen content of 89%. All chemical compounds were purchased from Sigma-Aldrich (United Kingdom) and were used as received. All NMR experiments were performed using a precursor solution of 100 mM [1-^13^C]disodium acetylenedicarboxylate, 6 mM [RuCp*(CH_3_CN)_3_]PF_6_ catalyst, and 200 mM sodium sulfite dissolved in D_2_O at 50 °C. It is been reported that sodium sulfite improves the selectivity of the trans hydrogenation reaction, although the mechanism of its action is not yet known.^33,60^

### Microfluidic Device

**Figure 2:**
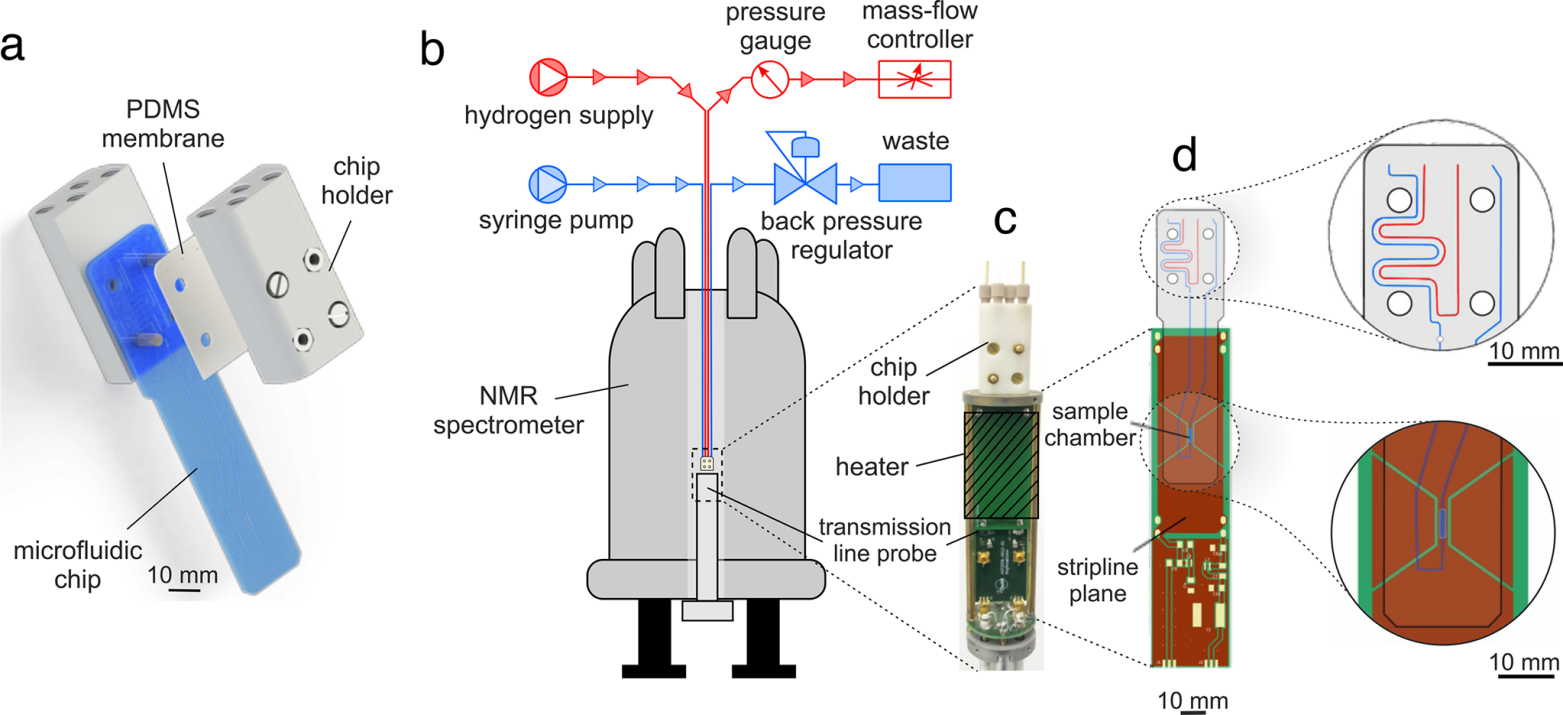
(a) Microfluidic chip assembly. (b) Schematic diagram of the experimental setup. (c) Transmission line probe with a heater indicated as the shaded area. (d) Drawing of the microfluidic device aligned with the stripline plane of the detector. The key areas of the drawing are enlarged.

The microfluidic device was made from three layers of polycarbonate (PC) (Self Adhesive Supplies, United Kingdom) with 0.25, 0.5, and 0.25 mm thicknesses for the top, middle, and bottom layers, respectively. The layers were cut from PC sheets by a LS3040 CO_2_ laser cutter (HPC Laser Ltd., United Kingdom) and were thermally bonded together as described elsewhere.^61^ A semipermeable polydimethylsiloxane (PDMS) membrane of 1 mm thickness (Shielding Solutions, United Kingdom) was placed over the top half of the chip to seal the chip and to allow hydrogen diffusion from the gas channel to the liquid channel. Figure 2a shows that the chip and the membrane were held together by 3D printed holders (ProtoLabs, United Kingdom) that attach threaded connectors for 1/16 in. capillaries (Cole-Parmer, United Kingdom) to the four access points on the chip for gas and liquid inlets and outlets.

In the magnet, the device was placed in a home-built transmission line probe as shown in Figure 2b. A heater was clamped outside of the stripline planes to heat the sample chamber in the microfluidic chip to 50 °C. This is indicated by the shaded area in Figure 2c. The heated area did not include the 3D printed holders so that the solution in contact with the hydrogen gas was kept at lower temperature in order to maximize the solubility of the hydrogen gas. The reaction products were detected in a 2.5 μL sample chamber. The chamber of the chip was aligned with the constrictions of the stripline detector as shown in Figure 2d.^21^

### Experimental Procedure

All experiments were performed inside the high-field NMR spectrometer as shown in Figure 2b. Experiments were conducted at 50 °C (at the sample chamber only) with the supply of hydrogen gas set to a pressure of 5 bar and flow rate of 10 mL  min^–1^, stabilized by a mass flow controller (Cole-Parmer, United Kingdom) connected at the end of the line. The flow of the precursor solution was controlled with a syringe pump (Cole-Parmer, United Kingdom) located outside the spectrometer. The target flow rate was set to 10 μL  min^–1^. Under these operating conditions, the NMR signal reached a steady-state after 10 min.

Proton singlet order in [1-^13^C]fumarate was converted into observable magnetization using the singlet-to-magnetization (S2M) pulse sequence shown in Figure 1e. Maximum efficiency was achieved using the following parameters: τ = 15.6 ms, *n*_2_ = 14, *n*_1_ = 7. The repetition delay was set to 60 s.

CW-S2M experiments were performed by applying continuous wave irradiation for 20 s at 0.5 and 2 kHz, while changing the resonance offset from 20 to −20 ppm. θ-S2M experiments were performed by applying a hard pulse of varying flip angle prior the S2M pulse sequence. This was achieved by varying the pulse duration from 0 to 8 μs in steps of 0.22 μs.

The reference spectrum was obtained using hydrogen in thermal equilibrium. The ^1^H  spectrum was obtained by applying a π/2 pulse and averaging over 400 scans with a recycle delay of 20 s.

## Results and Discussion

**Figure 3:**
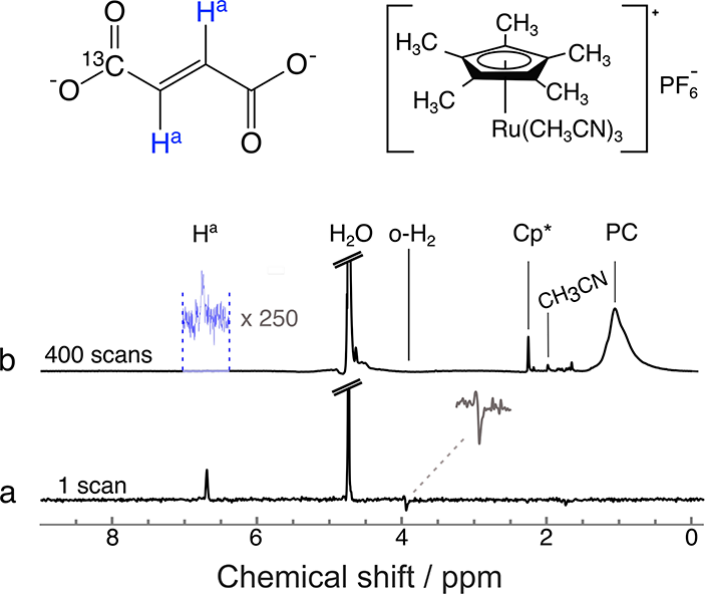
Steady state ^1^H NMR spectra of [1-^13^C]fumarate sample flowing at 10 μL min^–1^ in a microfluidic device. (a) Spectrum collected with the S2M pulse sequence with 89% parahydrogen. The trace displays a hyperpolarized [1-^13^C]fumarate peak at 6.6 ppm. The presence of exchanging hydrogen species is indicated at 4 ppm (o-H_2_). (b) Reference spectrum resulting from a π/2 pulse with hydrogen in thermal equilibrium. Cp*, catalyst methyl protons; PC, background signal from the polycarbonate chip material.

Figure 3a depicts a single-scan proton NMR spectrum obtained after application of the S2M pulse sequence in a steady-state flow experiment with 89% para-enriched H_2_. This can be compared to the 400-scan reference spectrum obtained after application of a π/2 pulse using hydrogen in thermal equilibrium (i.e., not para-enriched) in Figure 3b.

The spectra contain a peak at 6.6 ppm that corresponds to the fumarate protons H^a^. From the ratio of the signal intensity in the reference and hyperpolarized spectra, the ^1^H polarization was estimated. Accounting for the difference in the number of scans, the signal enhancement was calculated as 190 ± 10. At the field of 11.7 T and temperature of 50 °C, this corresponds to 0.7 ± 0.1% ^1^H polarization. At 10 μL  min^–1^ flow rate, the concentration of fumarate was 1.2 ± 0.5 mM, which corresponds to 1.2 ± 0.5% yield. This was calculated by comparing the intensity of the Cp* peak in the reference spectrum to the intensity of the fumarate peak and accounting for the difference in the number of protons.

The hyperpolarized spectrum features the aforementioned partially negative line at 4 ppm labeled o-H_2_. The heavy metal catalyst and dissolved molecular hydrogen form intermediate complexes where the two hydrogen nuclei occupy chemically inequivalent positions. At high magnetic field, this introduces a chemical shift difference between the two protons, which causes singlet state population to leak into the population of the central triplet state. In addition, the chemical shift difference lifts the degeneracy of the two triplet state transitions. In rapid exchange, this leads to a small partially negative line in the dissolved H_2_ signal,^46,47,54,57^ as displayed in the spectrum in Figure 3a.

**Figure 4:**
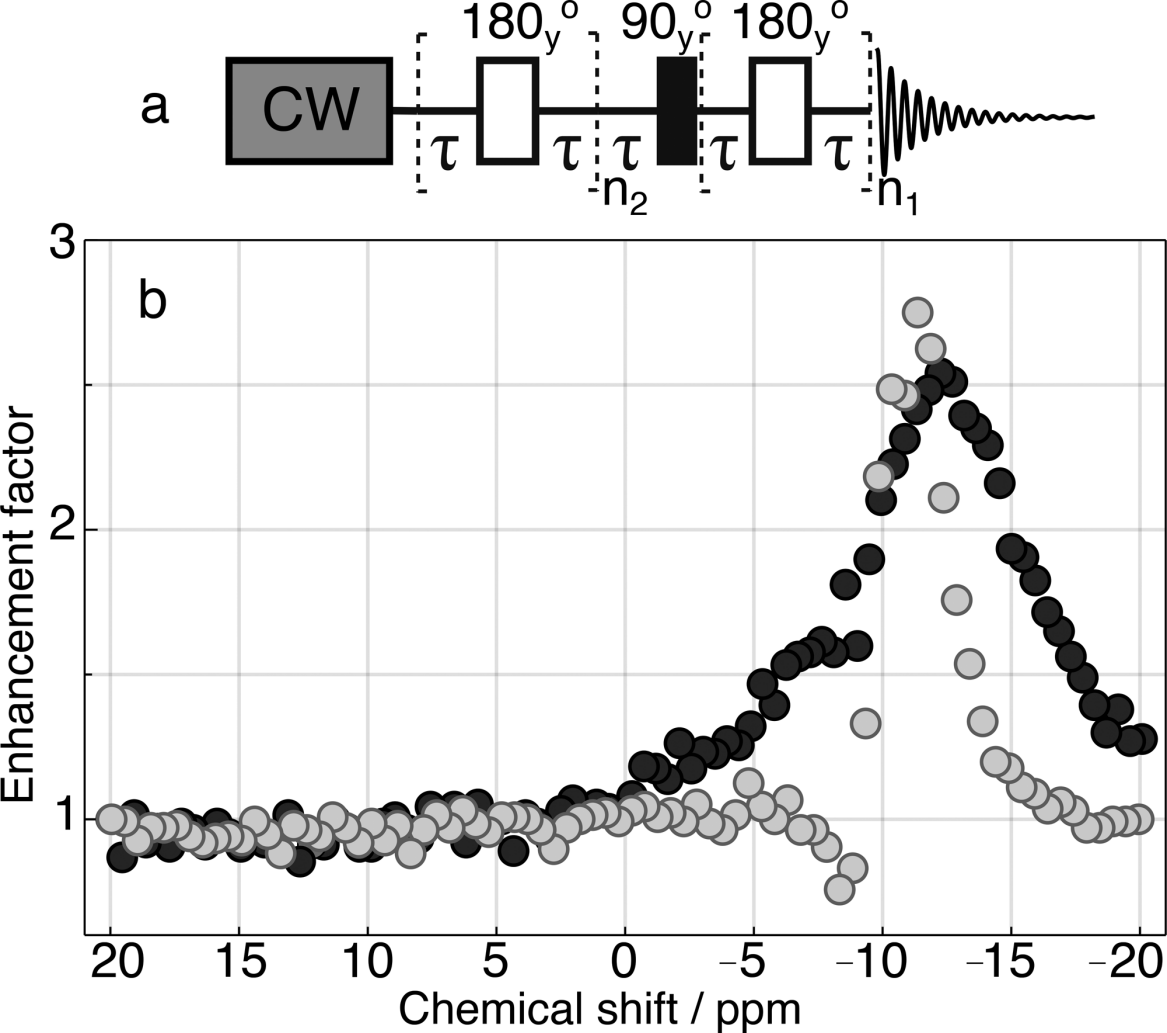
(a) Singlet-to-magnetization pulse sequence with spin-locking field applied during recycle delay. (b) Integral of signal intensity of the hyperpolarized protons of [1-^13^C]disodium fumarate as a function of the resonance offset of the spin-locking field. Experiments were preformed with two CW amplitudes, corresponding to 2 kHz and 0.5 kHz nutation frequency shown as black and gray data points, respectively. The signal amplitude was normalized to the signal acquired with CW frequency set to 20 ppm.

To suppress the effects of ST mixing, we performed experiments in which we applied continuous-wave (CW) irradiation to the sample for 20 s prior to the application of S2M and signal acquisition. The pulse sequence is shown in Figure 4a. The resulting integral of fumarate signal intensity at 6.6 ppm is plotted as a function of CW offset frequency in Figure 4b. Experiments were performed with two different CW amplitudes, corresponding to 0.5 kHz and 2 kHz nutation frequency on protons shown as gray and black circles, respectively.

The profiles of signal intensity against the CW irradiation frequency display a peak at around −11 ppm. This is a typical chemical shift of hydride species for ruthenium complexes,^62^ indicating that ST mixing does indeed occur for the hydride species and is suppressed by CW irradiation. The ^1^H spectra can be used to observe ST mixing, and this has been shown in the case of SABRE by either applying a single hard pulse after CW irradiation or a pulse sequence designed to probe higher spin-order if hydride species undergo very fast chemical exchange.^46,47^ In the present case, hydride species are not directly observable due to fast exchange and low sensitivity. The signal is enhanced by a factor of ∼3 when the spin-locking amplitude is set to either to 0.5 kHz or 2 kHz applied at −11 ppm. The peak width in each case corresponds roughly to the excitation bandwidth, resulting in a narrower peak at the lower CW amplitude.

We contrast this with another method, which has been used to address ST mixing effects: applying a hard pulse (which we will refer to as the purge pulse) to the protons prior to polarization transfer and signal acquisition. Application of a π/2 purge pulse on the proton channel depletes the |*T*_0_⟩ state, which partially reconstitutes the population difference between the |*S*_0_⟩ and |*T*_0_⟩ states.^46,52,55,57−59^

**Figure 5:**
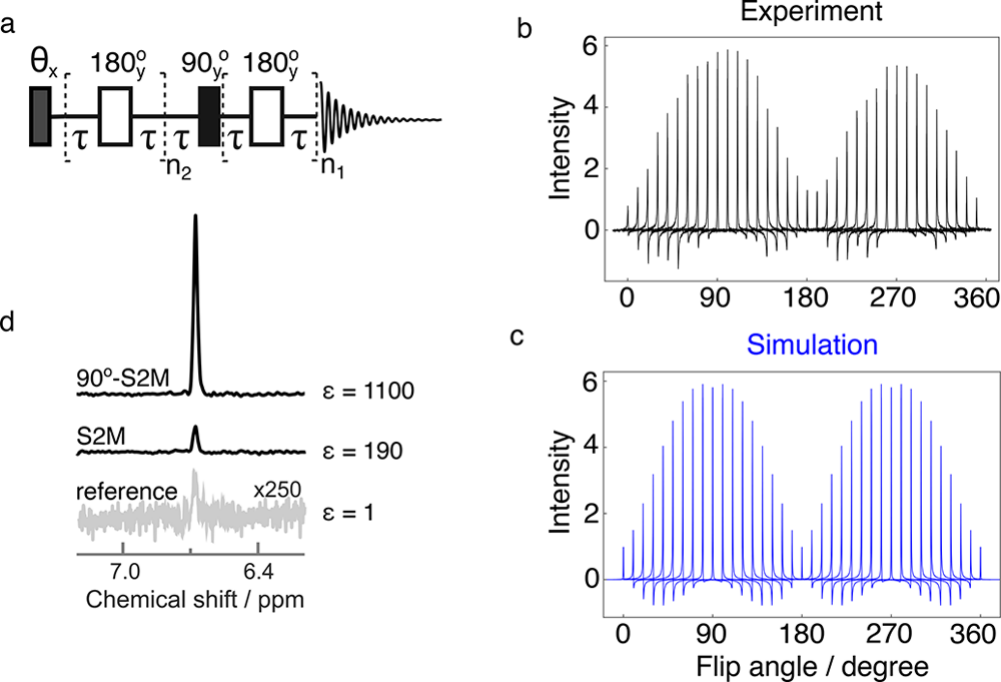
(a) θ-S2M pulse sequence. The θ angle was arrayed from 0° to 360° in steps of 10°. τ = 15.6 ms, *n*_1_ = 7, *n*_2_ = 14. (b) Experimentally obtained H^a^ signals of [1-^13^C]fumarate as a function of the purge pulse angle. The *y*-axis shows the improvement of the signal intensity normalized to the S2M signal without the purge pulse. (c) Computational simulation of the spin system using SpinDynamica software.^63^ (d) Comparison of the signal intensity of fumarate protons (H^a^) between the reference spectrum, pure S2M and 90°-S2M.

Figure 5a shows the pulse sequence used to investigate the phenomenon, and Figure 5b shows the hyperpolarized H^a^ proton signals obtained experimentally by varying the flip angle θ from 0° to 360° in steps of 10°. The signal shows an oscillatory dependence on the flip angle of the purge pulse, with maxima occurring at 90° and 270° and no improvement seen near 180°. The signal at 270° is about 15% less than at 90°. While this is likely due to *B*_1_ inhomogeneities, other factors, for example, chemical kinetics on the time scale of the pulse length, may also contribute.

The spectral peaks in Figure 5b also display phase distortions depending on the flip angle of the purge pulse. The origin of the effect was confirmed by numerical simulations using software package SpinDynamica,^63^ and the result is shown in Figure 5c. The simulation assumes that before the application of the sequence depicted in Figure 5a, the |*S*_0_⟩ and |*T*_0_⟩ states are 55% and 45% populated, respectively. The populations of the other triplet states are neglected. The agreement between experimental data and numerical simulation is striking. Both experiments and simulations show phase distortions when the flip angle is not an integer multiple of 90°. These phase distortions arise as follows: When the first pulse has a flip angle of 90°, the pulse transfers the population of the central triplet state |*T*_0_⟩ to the outer triplet states |*T*_±1_⟩, increasing the population difference between the singlet state |*S*_0_⟩ and the central triplet state |*T*_0_⟩ and hence enhancing the hyperpolarized NMR signal at the end of the pulse sequence. However, the flip angle of the first pulse is not a multiple of 90°, the transport of populations between the triplet state is accompanied by the excitation of single-quantum triplet–triplet coherences, of the form |*T*_±1_⟩⟨*T*_0_| and |*T*_0_⟩⟨*T*_±1_|. These coherences persist throughout the pulse sequence and appear as out-of-phase signal components in the observed spectrum, which have the effect of an undesirable phase shift of the observed peak.

In Figure 5d, a comparison is shown between the reference spectrum obtained with 400 scans and single scan NMR spectra of H^a^ protons after applying the S2M sequence with a purge pulse of 0° and 90°. The enhancement in the latter case was calculated to be 1100 ± 10 in contrast to 190 ± 10 without applying the purge pulse. This corresponds to 4.0 ± 0.1% ^1^H polarization and hence a nearly 6-fold improvement in achievable fumarate signal. The enhancement factor was calculated by comparing the integral of the H^a^ peak in the hyperpolarized and reference spectrum, accounting for the difference in the number of scans between the two spectra.

The hard-pulse method yielded a 6-fold improvement in the achievable fumarate signal, compared to 3-fold for the spin-locking method. This was unexpected since the spin-locking method can in principle lead to higher signal enhancements as it should mitigate the effect of ST mixing entirely. We believe the lower efficiency provided by spin locking is due to the micro-NMR probe design where the RF field is concentrated exclusively onto the sample chamber as shown in Figure 2d. Therefore, the solution outside the sample chamber is not affected by the RF irradiation, and thus the ST mixing cannot be suppressed for molecules of fumarate that formed in the channels before reaching the sample chamber. This is not a problem for the hard-pulse method since the pulse is applied after the chemical reaction.

The results obtained show the remarkable reproducibility and stability of the chemical reactions performed in the microfluidic device over the course of hours. A steady-state between the rate of chemical reaction to form the hyperpolarized product and the rate of relaxation was established, and without the confounding influence of these external factors it is possible to study and optimize pulse sequences in hyperpolarized NMR experiments. An additional benefit of working on a microfluidic scale is the small sample volumes required, meaning expensive or scarce samples can be more readily used. For example, the data in Figure 5b required 40 min of experimental time, consuming 400 μL of solution, which is the approximate volume required for a single PHIP experiment in a conventional 5 mm NMR tube.

The yield of fumarate in the chip was 1.2 ± 0.5%. The low yield of the reaction is most likely due to the limited uptake of hydrogen into the flowing solution. Finite element simulations of the chip have shown that when methanol is flowed through the chip at 10 μL  min^–1^ at a pressure of 5 bar, only 10 mM of hydrogen dissolves in the fluid.^64^ Since in this work water was used as the solvent, the concentration of hydrogen dissolved is expected to be lower due to poorer solubility of hydrogen in water. Modifications to the apparatus to improve the H_2_ uptake and yield of the reaction are currently underway.

## Conclusion

In this work we employed a microfluidic chip to run PHIP reactions, incorporating the hydrogenation, sample transport, RF excitation, and signal detection steps onto a single device. In the reaction, we hyperpolarized [1-^13^C]fumarate and used the S2M pulse sequence to generate in-phase proton magnetization for observation in the 2.5 μL sample chamber, achieving 4% proton polarization. We used this system to investigate pulsed NMR methods that reduce the detrimental effects of singlet–triplet mixing in this PHIP reaction. We showed that application of continuous wave irradiation prior to applying the S2M pulse sequence leads to a 3-fold improvement to the fumarate proton polarization and also allowed us to locate the chemical shift of the catalyst complex on which singlet–triplet mixing occurs. We contrasted that with application of a π/2 pulse prior to applying the S2M sequence, which led to a 6-fold improvement to the proton polarization.

Continuous-flow PHIP approach allows one to establish a constant stream of a hyperpolarized product, providing stable and reproducible conditions for the study of complex chemical and spin-dynamical phenomena in a well-controlled environment. This is an important step toward observation of metabolism in biological systems by hyperpolarized NMR on a single microfluidic device. By bringing hydrogen gas into solution through a membrane as opposed to bubbling or shaking, the chemical reaction is more stable and reaches a steady-state with a variation in the concentration of reaction product of 1%. By operating at a small volume-scale (microliters), the consumption of expensive materials is significantly reduced as compared to performing reactions in NMR tubes.

Not only does microfluidic implementation aid in the development of hyperpolarized NMR methods, but incorporating hyperpolarization to enhance NMR signals opens the door to the use of NMR as a detection method to study biological systems in microfluidic devices. Methods such as fluorescence spectroscopy require using specific fluorescent tags to track molecules, and UV–visible spectroscopy offers a limited ability to identify molecules. The molecular specificity and nondestructive nature of NMR spectroscopy makes it an ideal technique to track metabolic reactions, and direct production of hyperpolarized fumarate in a microfluidic chip is an important step toward this goal. However, further developments are required to make this dream a reality, such as the removal of toxic chemicals after the hyperpolarization process and the incorporation of ^13^C NMR for background-free detection with high chemical specificity and resolution.

Much work with hyperpolarized biomolecules relies on ^13^C hyperpolarization and detection, since this is preferable for *in vivo* imaging as the large background signals from water molecules are not present. The probe used for this work is doubly tuned for ^1^H  and ^13^C excitation and detection, but in order to perform such experiments, several issues need to be addressed. A prerequisite of using PHIP-polarized metabolites for biological studies is the ability to remove the catalyst and reaction side-products from the solution. This has been shown to be possible for [1-^13^C]fumarate via a precipitation procedure^32^ and for a variety of other PHIP-polarized metabolites via the side arm hydrogenation procedure.^55^ Precipitation procedures are not feasible in microfluidic devices as the solid would block the fluidic channels. However, scavenger compounds that bind the catalyst could potentially be used for this purpose.^65,66^
